# Albumin-Anchored Composite Ratios of Blood Urea Nitrogen, C-Reactive Protein, Lactate, and Creatinine for Predicting Mortality in Chronically Ill Intensive Care Unit Patients

**DOI:** 10.3390/jcm15072470

**Published:** 2026-03-24

**Authors:** Nilgün Şahin, Semih Aydemir, Nazan Has Selmi, İbrahim Ertaş, Yavuz Kutay Gökçe, Cihan Döğer, Gökçen Terzi, Mesher Ensarioğlu, Recep Dokuyucu

**Affiliations:** 1 Department of Anesthesiology and Reanimation, Ankara Guven Hospital, 06540 Ankara, Türkiye; 2 Department of Critical Care, Yıldırım Beyazıt University, Yenimahalle Training and Research Hospital, 06010 Ankara, Türkiye; semih.aydemir@saglik.gov.tr (S.A.); nazan.hasselmi@saglik.gov.tr (N.H.S.); 3 Department of Emergency Medicine, Yıldırım Beyazıt University, Yenimahalle Training and Research Hospital, 06010 Ankara, Türkiye; ibrahim.ertas1@saglik.gov.tr; 4 Department of Internal Medicine, Yıldırım Beyazıt University, Yenimahalle Training and Research Hospital, 06010 Ankara, Türkiye; yavuzkutay.gokce@saglik.gov.tr; 5 Department of Anesthesiology and Reanimation, Ankara City Hospital, 06800 Ankara, Türkiye; cihan.doger@sbu.edu.tr; 6 Department of Anesthesiology and Reanimation, Yıldırım Beyazıt University, Yenimahalle Training and Research Hospital, 06010 Ankara, Türkiye; gokcen.atilla@saglik.gov.tr; 7 Department of Anesthesiology and Reanimation, Health Sciences University Gülhane Training and Research Hospital, 06010 Ankara, Türkiye; mesher.ensarioglu@sbu.edu.tr; 8 Department of Physiology, Medical Specialty Training Center (TUSMER), 06420 Ankara, Türkiye; recep.dokuyucu@kocaelisaglik.edu.tr

**Keywords:** albumin-anchored ratios, critical care, mortality, BAR, CAR, LAR, ACR, prognostic biomarkers, chronic ICU patients

## Abstract

**Background:** This study aimed to evaluate the prognostic performance of four albumin-anchored ratios—blood urea nitrogen/albumin ratio (BAR), C-reactive protein/albumin ratio (CAR), lactate/albumin ratio (LAR), and albumin/creatinine ratio (ACR)—in predicting short-term mortality among intensive care unit (ICU) patients with pre-existing chronic comorbidities. Additionally, we assessed their incremental prognostic value beyond established severity scores such as APACHE II and SOFA. **Materials and Methods:** This retrospective cohort study included 520 chronically ill adult ICU patients admitted between July 2022 and July 2025. Patients with missing laboratory data, ICU stay <24 h, or postoperative monitoring only were excluded. BAR, CAR, LAR, and ACR were calculated from admission laboratory values. The primary outcome was 28-day mortality. Receiver operating characteristic (ROC) analyses, multivariate logistic regression, and model improvement metrics (C-statistics, NRI, IDI) were used to assess predictive performance. **Results:** Non-survivors had significantly higher BAR (15.0 vs. 8.2), CAR (39.2 vs. 19.1), and LAR (0.86 vs. 0.44) values and lower ACR (2.0 vs. 3.4) (all *p* < 0.001). In multivariate analysis, all four ratios independently predicted 28-day mortality (*p* < 0.001 for each). CAR showed the highest AUC (0.80), followed by LAR (0.79), BAR (0.78), and ACR (0.76). Incorporating all four ratios improved model discrimination (C-statistic 0.872 vs. 0.823; Δ = +0.049, *p* < 0.001) and reclassification (NRI = 0.162; IDI = 0.052). **Conclusions:** BAR, CAR, LAR, and ACR are independent and complementary predictors of short-term mortality in ICU patients with chronic comorbidities. Among them, CAR exhibited the best discriminative power. The combined use of these ratios enhanced risk prediction beyond traditional severity scores, suggesting their utility as simple, cost-effective markers for early mortality assessment. Because these indices are calculated from routinely measured laboratory parameters, they may represent practical and widely accessible tools for mortality risk stratification in routine ICU practice.

## 1. Introduction

Critically ill patients admitted to the intensive care unit (ICU) often have complex underlying chronic diseases that predispose them to adverse outcomes. Mortality risk in this population is influenced by a combination of baseline comorbidities, acute physiological derangements, and the host’s nutritional and inflammatory status. For the purposes of this study, chronically ill ICU patients were defined as individuals with at least one clinically significant chronic comorbidity affecting major organ systems [1,2,3,4,5]. Traditional severity scores such as the Acute Physiology and Chronic Health Evaluation II (APACHE II) and the Sequential Organ Failure Assessment (SOFA) provide valuable prognostic information but require multiple clinical and laboratory variables and may not be recalculated frequently in routine practice [6,7,8,9,10,11,12]. There is an ongoing need for simple, readily available biomarkers that can be obtained from routine laboratory testing and that integrate different aspects of pathophysiology.

Serum albumin is a negative acute phase reactant that reflects both nutritional status and systemic inflammation [13,14]. Hypoalbuminemia has been consistently associated with increased ICU mortality, prolonged hospital stays, and higher complication rates [15,16,17,18,19,20]. Ratios incorporating albumin with other laboratory parameters may provide more robust prognostic indicators by normalizing for the confounding influence of plasma volume shifts and nutritional depletion.

The blood urea nitrogen to albumin ratio (BUN/albumin ratio, BAR) has been proposed as a prognostic marker in various clinical settings, including community-acquired pneumonia, sepsis, and critical illness [17,21,22,23,24,25]. BAR combines the effects of catabolic state and renal function with the patient’s nutritional and inflammatory status, potentially improving mortality prediction compared to either parameter alone.

Similarly, the C-reactive protein to albumin ratio (CAR) has emerged as a composite index of inflammation and nutritional reserve [26,27,28,29,30,31]. Elevated CAR levels have been linked to poor outcomes in sepsis, acute coronary syndromes, and postoperative patients [32,33]. By integrating a sensitive inflammatory biomarker (CRP) with a nutritional marker (albumin), CAR may better capture the systemic inflammatory burden.

The lactate to albumin ratio (LAR) has also attracted attention as a prognostic indicator, especially in septic and postoperative populations [34,35,36,37,38,39,40,41]. Elevated lactate levels reflect tissue hypoperfusion and anaerobic metabolism, while concurrent hypoalbuminemia may signify worse physiological reserve; together, these factors may synergistically worsen prognosis [34,35,37,40,41].

The albumin to creatinine ratio (ACR) is traditionally used in nephrology as a marker of renal injury and endothelial dysfunction [42,43,44]. In critical care, both urinary and serum-based ACR measurements may serve as indicators of multiorgan stress and microvascular damage [45,46]. In chronic disease patients admitted to the ICU, impaired renal function and hypoalbuminemia frequently coexist, potentially making this ratio a useful risk stratification tool.

Despite the individual prognostic significance of BAR, CAR, LAR, and ACR, there is a paucity of studies directly comparing these ratios in the same cohort of chronically ill ICU patients. Furthermore, their incremental predictive value when added to established severity scores has not been fully elucidated. Understanding which of these indices best predicts mortality, and whether they complement existing risk models, could enhance early risk stratification and guide resource allocation in this high-risk population.

The present study aimed to evaluate the prognostic performance of BAR, CAR, LAR, and ACR for predicting short-term mortality in chronically ill patients admitted to the ICU. Additionally, we sought to determine whether these ratios provide incremental prognostic value beyond APACHE II and SOFA scores.

## 2. Materials and Methods

### 2.1. Study Design and Setting

This retrospective observational cohort study was conducted in the adult Intensive Care Unit (ICU) of Ankara City Hospital, a tertiary referral center with a capacity of 700 ICU beds, between 1 July 2022, and 30 July 2025. The ICU admits patients with a wide range of medical and surgical conditions, including sepsis and septic shock. The study was designed and reported in accordance with the Strengthening the Reporting of Observational Studies in Epidemiology (STROBE) guidelines. In the present study, chronically ill ICU patients were defined as individuals with at least one documented chronic comorbidity such as chronic kidney disease, chronic obstructive pulmonary disease, congestive heart failure, chronic liver disease, malignancy, or diabetes mellitus.

All consecutive adult patients (≥18 years) admitted to the ICU during the study period were screened for eligibility. Patients were included if they had at least one documented chronic comorbidity, including chronic kidney disease, chronic obstructive pulmonary disease, congestive heart failure, chronic liver disease, malignancy, or diabetes mellitus. Hypertension alone was not used as a defining inclusion criterion due to its high prevalence in the general adult population and its relatively limited impact on ICU admission severity. In this study, patients fulfilling this criterion were operationally defined as chronically ill ICU patients, and if serum blood urea nitrogen (BUN), creatinine, albumin, C-reactive protein (CRP), and lactate levels were available within the first 6 h of ICU admission. Patients were excluded if their ICU length of stay was less than 24 h, if they were admitted for routine postoperative monitoring without complications, if they were pregnant, if they were receiving extracorporeal membrane oxygenation (ECMO) at the time of admission, or if any of the key laboratory parameters required to calculate the studied ratios were missing. Patients with missing key laboratory parameters required for the calculation of the biomarker ratios (BUN, creatinine, albumin, CRP, or lactate) were excluded from the analysis. The proportion of missing laboratory data was low and mainly related to incomplete measurements at ICU admission.

### 2.2. Data Collection and Outcome Measures

Demographic, clinical, and laboratory data were obtained from the hospital’s electronic medical record system. The recorded variables included demographic characteristics (age, sex, and body mass index [BMI]) and clinical information such as primary diagnosis, comorbidities, presence of sepsis or septic shock according to Sepsis-3 criteria, need for mechanical ventilation, vasopressor therapy, renal replacement therapy (RRT), ICU length of stay, hospital length of stay, and survival status at 28 days, at hospital discharge, and at 90 days. Severity of illness was assessed using the Acute Physiology and Chronic Health Evaluation II (APACHE II) and Sequential Organ Failure Assessment (SOFA) scores, both calculated within the first 24 h of ICU admission. Laboratory parameters included serum blood urea nitrogen (BUN, mg/dL), creatinine (mg/dL), albumin (g/dL), C-reactive protein (CRP, mg/L), and lactate (mmol/L). The primary indication for ICU admission was also recorded and categorized according to the principal clinical diagnosis at the time of ICU admission (e.g., sepsis/septic shock, acute respiratory failure, cardiac causes, neurological disorders, or other medical conditions).

The following ratios were calculated for each patient:

❖ BUN/Albumin ratio (BAR): BUN (mg/dL)/Albumin (g/dL).
❖ CRP/Albumin ratio (CAR): CRP (mg/L)/Albumin (g/dL).
❖ Lactate/Albumin ratio (LAR): Lactate (mmol/L)/Albumin (g/dL).
❖ Albumin/Creatinine ratio (A/C): Albumin (g/dL)/Creatinine (mg/dL).

**Figure 1:**
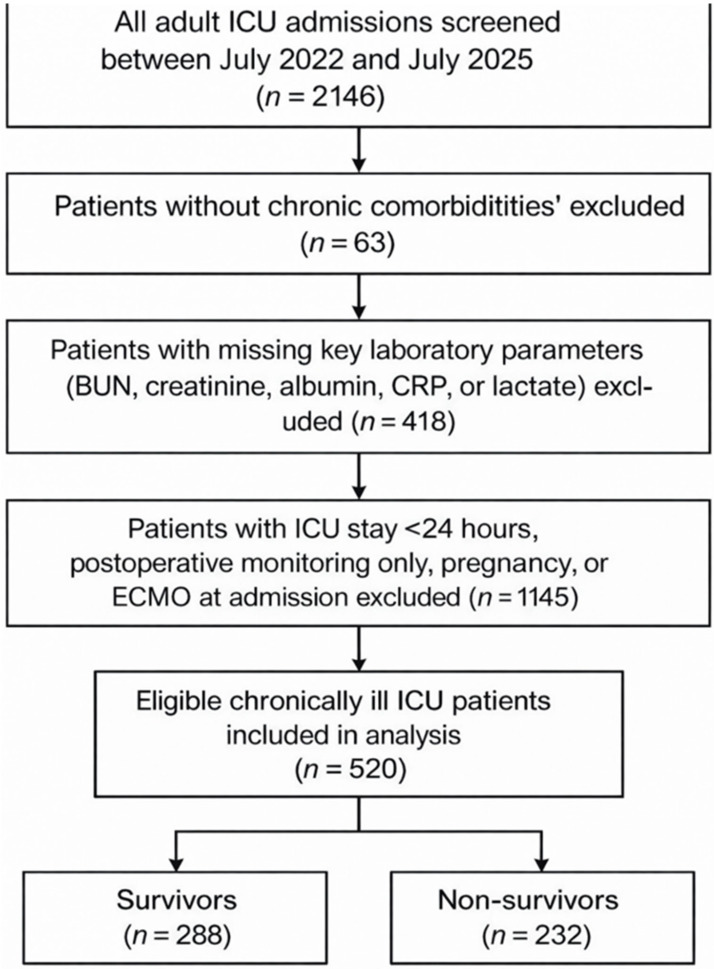
Flowchart of study.

All laboratory analyses were performed in the hospital’s central laboratory, accredited according to ISO 15189 standards [47], using standardized biochemical assays. The primary outcome of the study was 28-day all-cause mortality following ICU admission. Secondary outcomes included in-hospital mortality, 90-day mortality, ICU length of stay, and hospital length of stay (Figure 1).

### 2.3. Statistical Analysis

All statistical analyses were performed using IBM SPSS Statistics for Windows, version 27.0 (IBM Corp., Armonk, NY, USA) and R software, version 4.3.2 (R Foundation for Statistical Computing, Vienna, Austria). The Shapiro–Wilk test was used to assess the normality of distribution for continuous variables. Normally distributed variables were expressed as mean ± standard deviation (SD), whereas non-normally distributed variables were presented as median with interquartile range (IQR). Categorical variables were summarized as frequencies and percentages. Group comparisons were conducted using the independent-samples *t*-test or Mann–Whitney U test for continuous variables and the chi-square test or Fisher’s exact test for categorical variables. Receiver operating characteristic (ROC) curve analysis was performed to evaluate the predictive performance of the blood urea nitrogen/albumin ratio (BAR), C-reactive protein/albumin ratio (CAR), lactate/albumin ratio (LAR), and albumin/creatinine ratio (A/C) for 28-day mortality, with the area under the curve (AUC) and 95% confidence intervals (CIs) calculated. AUCs were compared using the DeLong test, and optimal cut-off values were determined by Youden’s index. Multivariate logistic regression analysis was used to assess the independent association of each ratio with 28-day mortality after adjusting for age, sex, APACHE II score, SOFA score, and other significant univariate predictors, with odds ratios (ORs) and 95% CIs reported. Model performance was further evaluated using C-statistics, net reclassification improvement (NRI), integrated discrimination improvement (IDI), and calibration plots with 1000 bootstrap resamples. Kaplan–Meier survival curves were generated for quartiles of each biomarker ratio, and differences were assessed using the log-rank test. In addition, the predictive performance of the APACHE II and SOFA scores was evaluated using ROC curve analysis and compared with the albumin-based ratios using the DeLong test. A two-tailed *p*-value < 0.05 was considered statistically significant.

## 3. Results

The most common indications for ICU admission were sepsis or septic shock, followed by acute respiratory failure and cardiac causes. Neurological conditions and other medical disorders accounted for a smaller proportion of admissions. Baseline demographic, clinical, and laboratory characteristics of the study population according to 28-day survival status were shown in Table 1. Non-survivors were significantly older (72 [64–79] vs. 65 [55–73] years, *p* < 0.001) and more frequently presented with sepsis or septic shock (50.9% vs. 31.9%, *p* < 0.001). Chronic comorbidities, including chronic kidney disease, congestive heart failure, chronic liver disease, and malignancy, were markedly more prevalent among non-survivors. The need for mechanical ventilation (78.4% vs. 41.0%), vasopressor support (72.4% vs. 29.2%), and renal replacement therapy (29.3% vs. 7.6%) was also significantly higher in patients who died (*p* < 0.001 for all) (Table 1).

Severity scores, laboratory parameters, and calculated ratios according to 28-day survival status were shown in Table 2. Non-survivors exhibited significantly higher disease severity, as reflected by both APACHE II (23 [19–27] vs. 18 [15–21], p < 0.001) and SOFA scores (9 [7–12] vs. 6 [4–8], p < 0.001). In terms of biochemical parameters, non-survivors had markedly elevated blood urea nitrogen (42 vs. 28 mg/dL), creatinine (1.40 vs. 1.00 mg/dL), C-reactive protein (110 vs. 65 mg/L), and lactate (2.4 vs. 1.5 mmol/L) levels compared to survivors (*p* < 0.001 for all). Conversely, serum albumin was significantly lower in the non-survivor group (2.8 vs. 3.4 g/dL, *p* < 0.001), reflecting a combined state of malnutrition and systemic inflammation. The BUN/albumin ratio (15.0 vs. 8.2), CRP/albumin ratio (39.2 vs. 19.1), and lactate/albumin ratio (0.86 vs. 0.44) were substantially higher in patients who died, whereas the albumin/creatinine ratio was significantly lower (2.0 vs. 3.4; *p* < 0.001) (Table 2).

**Figure 2:**
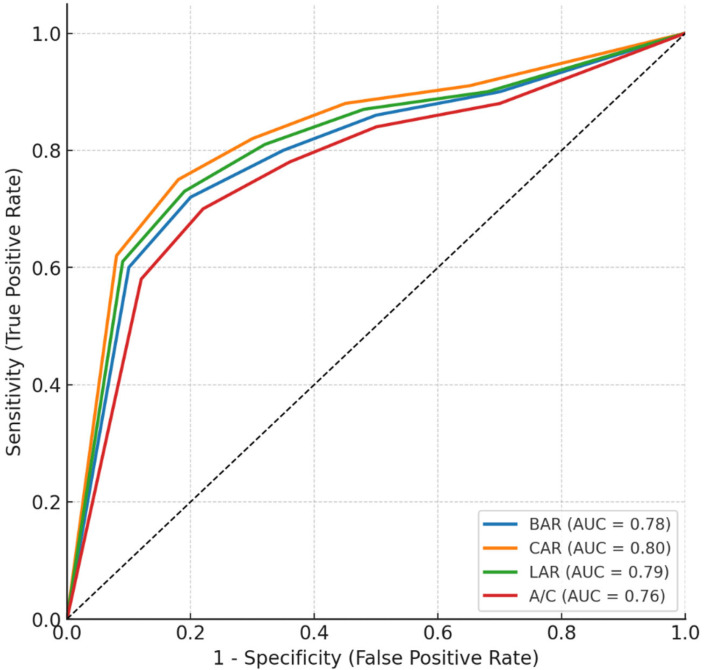
ROC curves for BAR, CAR, LAR, and A/C in predicting 28-day mortality.

ROC curve analysis of BAR, CAR, LAR, and A/C for predicting 28-day mortality was shown in Table 3. All four ratios demonstrated statistically significant discriminatory ability (*p* < 0.001 for each). Among them, the CAR showed the highest area under the curve (AUC = 0.80, 95% CI: 0.76–0.84), followed closely by the LAR (AUC = 0.79, 95% CI: 0.75–0.83), the BAR (AUC = 0.78, 95% CI: 0.74–0.82), and the ACR (AUC = 0.76, 95% CI: 0.72–0.80). Although CAR exhibited the best overall performance, the pairwise AUC comparisons using the DeLong test revealed no statistically significant differences between CAR and BAR (*p* = 0.18) or CAR and LAR (*p* = 0.24). However, the ACR showed slightly lower discriminative power, differing significantly from CAR (*p* = 0.03). The optimal cut-off values determined by Youden’s index were 11.5 for BAR, 28.0 for CAR, 0.65 for LAR, and 2.6 for ACR. Sensitivity and specificity values ranged between 70 and 77%. To contextualize the predictive performance of albumin-based biomarkers, ROC analyses were also performed for the APACHE II and SOFA scores. The APACHE II score demonstrated an AUC of 0.82 (95% CI: 0.78–0.85), while the SOFA score showed an AUC of 0.80 (95% CI: 0.76–0.84) for predicting 28-day mortality. These values were comparable to the predictive performance of the albumin-based ratios, particularly CAR (AUC = 0.80) and LAR (AUC = 0.79). However, when combined with the severity scores, the albumin-based biomarkers significantly improved model discrimination, as reflected by the higher C-statistic observed in the extended model (Model-2) (Table 3, Figure 2).

Multivariate logistic regression analysis of biomarker ratios for prediction of 28-day mortality was shown in Table 4. Specifically, each one-unit increase in the BAR was associated with a 7% higher risk of death (adjusted OR: 1.07, 95% CI: 1.04–1.10), while each one-unit increase in the CAR increased the risk by 2% (OR: 1.02, 95% CI: 1.01–1.03). Similarly, a 0.1-unit increase in the LAR was linked to an 8% higher mortality risk (OR: 1.08, 95% CI: 1.04–1.12). In contrast, each one-unit decrease in the ACR corresponded to a 21% increase in the odds of death (OR: 1.21, 95% CI: 1.10–1.33). Age and higher APACHE II and SOFA scores were also independently associated with mortality (Table 4).

**Figure 3:**
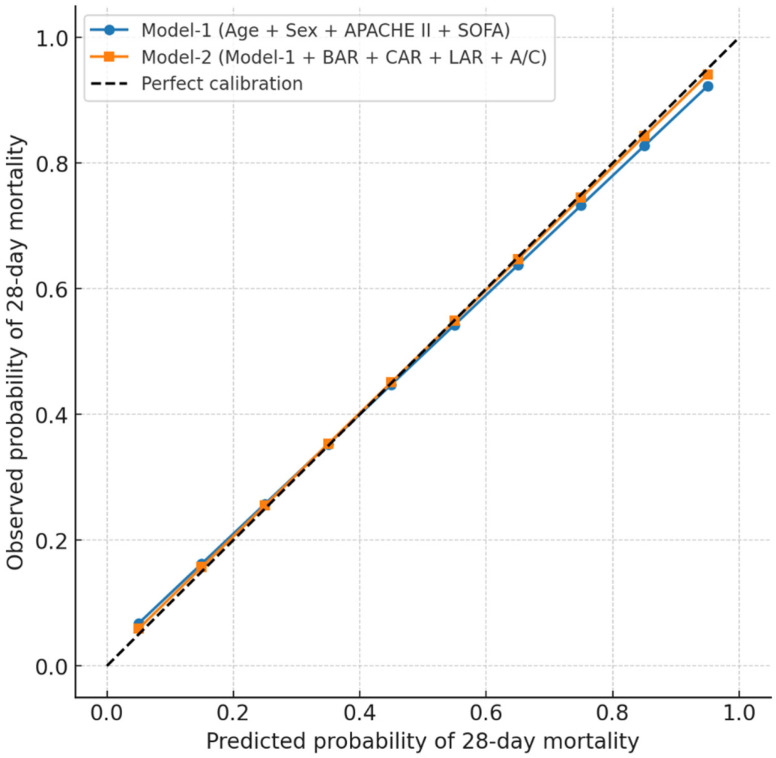
Calibration curves for Model-1 and Model-2.

Incremental prognostic value of biomarker ratios beyond baseline severity scores for 28-day mortality prediction was shown in Table 5. The extended model (Model-2) significantly improved overall predictive performance for 28-day mortality compared to the baseline model (Model-1). Specifically, the C-statistic increased from 0.823 (95% CI: 0.794–0.852) in Model-1 to 0.872 (95% CI: 0.847–0.897) in Model-2 (Δ = +0.049, *p* < 0.001). Furthermore, both continuous and categorical NRI values were positive and statistically significant (0.162 and 0.141, respectively; *p* < 0.001 for both). The IDI also increased by 0.052 (*p* < 0.001), reflecting a better separation between survivors and non-survivors. Additionally, the Brier score decreased from 0.158 to 0.142 (Table 5, Figure 3).

**Figure 4:**
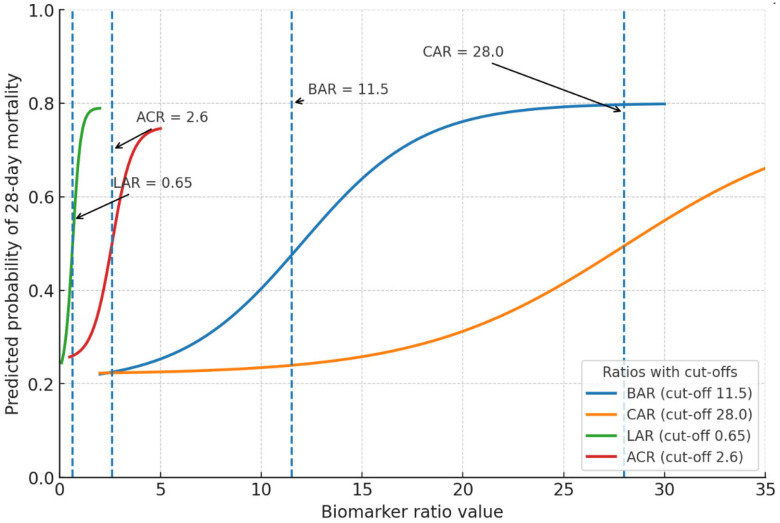
Restricted cubic spline (RCS) curves for biomarker ratios.

RCS curves for biomarker ratios were shown in Figure 4. For the BAR, mortality risk began to rise significantly beyond approximately 10 units, with a steep upward slope above 15, reflecting the combined effect of renal dysfunction and hypoalbuminemia on poor outcomes. The CAR showed a nearly linear increase in mortality risk across its range, emphasizing the strong contribution of systemic inflammation and nutritional depletion to critical illness severity. The LAR displayed a pronounced positive association with mortality, with risk escalating markedly beyond 0.6, consistent with increasing tissue hypoxia and metabolic stress. In contrast, the ACR demonstrated an inverse, curvilinear pattern, where lower values (<2.5) were strongly associated with higher mortality (Figure 4).

## 4. Discussion

This study comprehensively evaluated the prognostic significance of four albumin-anchored ratios—BAR, CAR, LAR, and ACR—in predicting 28-day mortality among chronically ill ICU patients. The principal finding was that all four ratios were independently associated with short-term mortality, even after adjustment for age, sex, and established severity scores (APACHE II and SOFA). Moreover, the combination of these ratios improved the discriminative ability of baseline models, as reflected by a significant increase in C-statistic, NRI, and IDI.

The prognostic role of BAR has been increasingly recognized in critical care. Xie et al. found that elevated BAR was a strong predictor of in-hospital mortality in ICU patients with atrial fibrillation [21]. Bayrakci and Eygi demonstrated that BAR predicted adverse outcomes in elderly ICU patients with acute kidney injury, while Li et al. confirmed its prognostic value in acute respiratory failure [22,23]. Our results align with these findings, showing a significant association between higher BAR and 28-day mortality (AUC = 0.78, *p* < 0.001).

Unlike studies focusing on specific disease groups, our cohort included patients with diverse chronic comorbidities, suggesting that BAR reflects a universal interaction between catabolic state, renal dysfunction, and nutritional depletion. Hang et al. and Min et al. proposed that BAR integrates renal stress with systemic inflammation, reinforcing its utility as a readily available bedside index [24,25].

CAR has been validated in multiple clinical settings as a robust marker of inflammation–nutrition imbalance. Son et al. showed that CAR correlated with cancer prognosis, while Jeon et al. reported that elevated CAR independently predicted mortality in acute kidney injury, requiring continuous renal replacement therapy [26,30]. In line with these reports, our study found CAR to be the single best-performing ratio (AUC = 0.80), outperforming other indices although not significantly higher than BAR or LAR (*p* > 0.05, DeLong). Lucijanic et al. and Sines et al. also demonstrated the prognostic relevance of CAR in systemic inflammatory conditions such as COVID-19, underscoring its broad clinical applicability [27,28]. The integration of CRP, a sensitive acute-phase reactant, with albumin, a negative acute-phase reactant, captures both the magnitude and chronicity of inflammation—a mechanism consistent with the observed incremental predictive improvement in our cohort.

The relatively higher predictive performance of CAR compared with the other ratios may be explained by the central role of systemic inflammation in critical illness. CRP is a highly sensitive marker of acute inflammatory activation and has been strongly associated with sepsis severity, organ dysfunction, and mortality. At the same time, albumin reflects both nutritional status and the systemic inflammatory response. The combination of a positive acute-phase reactant (CRP) and a negative acute-phase reactant (albumin) may therefore provide a more integrated reflection of the inflammatory burden and physiological reserve of critically ill patients.

In contrast, other ratios included in this study capture more specific aspects of pathophysiology. For example, BAR primarily reflects renal dysfunction and catabolic stress, while LAR is closely related to tissue hypoperfusion and metabolic stress. ACR, on the other hand, reflects the interaction between renal function and nutritional status. Because systemic inflammation is a central driver of organ dysfunction in many ICU conditions—including sepsis, shock, and multiorgan failure—the inflammatory component captured by CAR may explain its relatively stronger prognostic performance in our cohort.

Our study found that LAR (AUC = 0.79) was an independent predictor of mortality after adjusting for APACHE II and SOFA scores. This is concordant with findings by Bou Chebl et al., who first described LAR as a reliable marker of sepsis severity and in-hospital mortality [35]. Acharya et al. and Le Xuan et al. further validated LAR’s prognostic accuracy in sepsis-associated organ dysfunction [36]. The pathophysiological rationale lies in the synergistic effect of tissue hypoperfusion (lactate) and systemic malnutrition (albumin). Wang et al. confirmed that LAR closely correlates with both hepatic dysfunction and sepsis outcomes [37]. Our findings extend these results to a chronically ill ICU population, suggesting that LAR captures metabolic stress beyond acute sepsis and can be interpreted as a marker of limited physiological reserve.

The inverse association between ACR and mortality observed in our study (AUC = 0.76) supports emerging evidence linking impaired renal–nutritional balance with poor outcomes. Lower ACR values primarily reflect the coexistence of hypoalbuminemia and elevated serum creatinine, both of which are common in critically ill patients. Hypoalbuminemia may result from systemic inflammation, increased capillary permeability, and reduced hepatic protein synthesis, whereas elevated creatinine reflects renal dysfunction and impaired metabolic clearance. The coexistence of these two processes may therefore indicate a state of multiorgan physiological stress, which has been strongly associated with adverse outcomes in ICU populations.

In this context, a low ACR can be interpreted as a composite indicator of both reduced nutritional reserve and impaired renal function, providing a plausible biological explanation for its association with increased mortality. Hu et al. found that lower serum ACR predicted higher mortality in patients with sepsis, while Sun et al. reported a similar association in ischemic stroke patients [43,44]. Our findings are consistent with these reports and suggest that hypoalbuminemia relative to creatinine elevation reflects both reduced protein synthesis and declining renal clearance capacity. Unlike traditional urinary ACR, serum-based measurements are easier to obtain and may be more stable in ICU settings, offering a practical clinical advantage. However, the slightly lower discriminative ability of ACR compared with CAR or LAR suggests that renal-specific stress may play a secondary role relative to systemic inflammatory or metabolic stress in chronically ill ICU patients.

In addition to renal dysfunction and hypoalbuminemia, another possible explanation for the inverse association between ACR and mortality may relate to frailty and sarcopenia. Serum creatinine levels are influenced by skeletal muscle mass; therefore, chronically ill or frail patients with reduced muscle mass may exhibit relatively low creatinine concentrations. In such cases, alterations in ACR may partly reflect the combined effects of systemic inflammation, malnutrition, and muscle wasting, which are common in critically ill and chronically diseased populations. Previous studies have shown that sarcopenia and frailty are independently associated with increased mortality in ICU patients. Thus, the relationship between ACR and mortality may not solely represent renal dysfunction but may also capture broader aspects of metabolic reserve and physiological vulnerability in chronically ill patients.

Importantly, adding all four albumin-anchored ratios to the baseline model (age + sex + APACHE II + SOFA) significantly improved predictive performance (C-statistic = 0.872 vs. 0.823; *p* < 0.001). This finding is consistent with the concept of “multidimensional metabolic stress”, whereby combined indices better represent the interplay among inflammation, catabolism, and organ dysfunction. Similar model improvements have been noted by Li et al. for BAR and Jeon et al. for CAR when added to classical ICU scores, suggesting that these ratios offer additive information not captured by APACHE II or SOFA alone [23,30]. Furthermore, our restricted cubic spline analysis revealed dose–response associations for all ratios, supporting their continuous predictive validity rather than a simple dichotomous threshold.

The pathophysiological explanation for our findings lies in the integrative role of albumin as both a nutritional and anti-inflammatory marker. Hypoalbuminemia potentiates endothelial dysfunction, capillary leakage, and reduced antioxidant capacity [13,15,16,17,18,20,41]. When combined with markers of renal (BUN, creatinine), inflammatory (CRP), or metabolic (lactate) stress, albumin-anchored ratios can delineate distinct dimensions of critical illness. Clinically, these ratios can be computed from standard laboratory panels without additional cost, making them ideal for early triage or serial monitoring in resource-limited settings. Their incorporation into electronic ICU dashboards could enable dynamic risk assessment complementary to APACHE II and SOFA.

When compared with traditional severity scores, the albumin-anchored ratios demonstrated a comparable predictive ability. While APACHE II and SOFA remain well-validated prognostic tools in critical care, they require multiple physiological variables and may be recalculated less frequently in clinical practice. In contrast, albumin-based ratios can be derived from routine laboratory tests and may provide rapid bedside risk stratification. The improved discrimination observed when these biomarkers were added to the baseline model suggests that they capture additional aspects of metabolic stress and systemic inflammation not fully reflected by conventional severity scores.

The major strengths of this study include a large, heterogeneous cohort of chronically ill ICU patients, comprehensive statistical adjustment, and the simultaneous comparison of four novel ratios. However, some limitations should be acknowledged. The retrospective design may introduce residual confounding. Although our models were adjusted for disease severity using APACHE II and SOFA scores, additional clinical factors such as nutritional status, systemic inflammation, and hemodynamic instability requiring vasopressor therapy may also influence albumin and lactate levels and cannot be completely accounted for. Second, the study was conducted in a single center and limited to patients with chronic comorbidities, which may restrict the generalizability of the findings to other ICU populations. Third, serial measurements of the studied biomarkers were not available, and therefore dynamic changes over time could not be evaluated. Another limitation of this study is the exclusion of patients with missing laboratory data. Although the proportion of missing data was relatively small, this approach may introduce selection bias. However, multiple imputation was not applied because the studied biomarkers were directly derived from the missing laboratory variables, and imputation could artificially alter the calculated ratios. Finally, although the study period spanned several years (2022–2025), ICU management protocols at our institution remained largely consistent and followed established critical care guidelines. Nevertheless, minor variations in clinical practice over time cannot be completely excluded. Despite these limitations, the strong and independent associations observed support the clinical utility of these ratios in mortality prediction.

## 5. Conclusions

In conclusion, BAR, CAR, LAR, and ACR are independent and complementary predictors of short-term mortality in ICU patients with chronic comorbidities. Among them, CAR demonstrated the highest discriminative power, while the combined use of all four ratios significantly enhanced prognostic accuracy beyond established severity scores. These biomarkers are derived from routinely available laboratory parameters and therefore represent low-cost and easily accessible tools that may support early risk stratification in daily ICU practice. Future multicenter, prospective studies should explore dynamic trends and integrate these biomarkers into composite predictive models for personalized critical care management.

## Figures and Tables

**Table 1:** Baseline demographic, clinical, and laboratory characteristics of the study population according to 28-day survival status.

Variable	Survivors (*n* = 288)	Non-Survivors (*n* = 232)	*p*-Value
	*n* (%) or Median [Interquartile Range]		
ICU admission indication			
Age (years)	65 [55–73]	72 [64–79]	<0.001
Male sex, *n* (%)	160 (55.6)	140 (63.1)	0.14
BMI (kg/m^2^)	26.1 [24.2–28.0]	25.8 [23.5–27.5]	0.09
Primary diagnosis—sepsis/septic shock	92 (31.9)	118 (50.9)	<0.001
Chronic kidney disease	54 (18.8)	72 (31.0)	0.002
Chronic obstructive pulmonary disease	38 (13.2)	46 (19.8)	0.05
Congestive heart failure	40 (13.9)	55 (23.7)	0.003
Chronic liver disease	18 (6.3)	26 (11.2)	0.04
Malignancy	22 (7.6)	34 (14.7)	0.01
Diabetes mellitus	82 (28.5)	79 (34.1)	0.18
Mechanical ventilation	118 (41.0)	182 (78.4)	<0.001
Vasopressor therapy	84 (29.2)	168 (72.4)	<0.001
Renal replacement therapy	22 (7.6)	68 (29.3)	<0.001
Lactate/Albumin ratio (LAR)	0.44 [0.33–0.61]	0.86 [0.64–1.20]	<0.001
Albumin/Creatinine ratio (A/C)	3.4 [2.7–4.2]	2.0 [1.5–2.5]	<0.001

**Table 2:** Severity scores, laboratory parameters, and calculated ratios according to 28-day survival status.

Variable	Survivors (*n* = 288)	Non-Survivors (*n* = 232)	*p*-Value
	*n* (%) or Median [Interquartile Range]		
APACHE II score	18 [15–21]	23 [19–27]	<0.001
SOFA score	6 [4–8]	9 [7–12]	<0.001
BUN (mg/dL)	28 [20–37]	42 [32–58]	<0.001
Creatinine (mg/dL)	1.00 [0.80–1.20]	1.40 [1.10–1.80]	<0.001
Albumin (g/dL)	3.4 [3.0–3.7]	2.8 [2.4–3.2]	<0.001
CRP (mg/L)	65 [40–105]	110 [72–162]	<0.001
Lactate (mmol/L)	1.5 [1.2–2.0]	2.4 [1.9–3.2]	<0.001
BUN/Albumin ratio (BAR)	8.2 [6.0–11.0]	15.0 [10.6–20.5]	<0.001
CRP/Albumin ratio (CAR)	19.1 [11.4–32.5]	39.2 [25.0–63.8]	<0.001
Lactate/Albumin ratio (LAR)	0.44 [0.33–0.61]	0.86 [0.64–1.20]	<0.001
Albumin/Creatinine ratio (A/C)	3.4 [2.7–4.2]	2.0 [1.5–2.5]	<0.001

**Table 3:** ROC curve analysis of BAR, CAR, LAR, and A/C for predicting 28-day mortality.

Biomarker	AUC (95% CI)	Cut-Off	Sensitivity (%)	Specificity (%)	*p*-Value	DeLong *p*-Value *
BAR	0.78 (0.74–0.82)	11.5	74.6	71.5	<0.001	—
CAR	0.80 (0.76–0.84)	28.0	77.2	73.1	<0.001	0.18 (vs. BAR)
LAR	0.79 (0.75–0.83)	0.65	75.0	72.2	<0.001	0.24 (vs. CAR)
ACR	0.76 (0.72–0.80)	2.6	70.3	69.4	<0.001	0.03 (vs. CAR)

BUN/Albumin ratio: BAR, CRP/Albumin ratio: CAR, Lactate/Albumin ratio: LAR, Albumin/Creatinine ratio: ACR. * DeLong *p*-value: comparison of AUC between the given biomarker and the highest AUC biomarker (CAR).

**Table 4:** Multivariate logistic regression analysis of biomarker ratios for prediction of 28-day mortality *.

Variable	Adjusted OR	95% CI	*p*-Value
Age (per 1-year increase)	1.03	1.01–1.05	0.002
Male gender	1.18	0.82–1.71	0.37
APACHE II score (per 1-point increase)	1.12	1.08–1.16	<0.001
SOFA score (per 1-point increase)	1.15	1.09–1.21	<0.001
BUN/Albumin ratio (BAR) (per 1-unit increase)	1.07	1.04–1.10	<0.001
CRP/Albumin ratio (CAR) (per 1-unit increase)	1.02	1.01–1.03	<0.001
Lactate/Albumin ratio (LAR) (per 0.1-unit increase)	1.08	1.04–1.12	<0.001
Albumin/Creatinine ratio (ACR) (per 1-unit decrease)	1.21	1.10–1.33	<0.001

* Model adjusted for age, sex, APACHE II score, SOFA score, and all four biomarker ratios. OR: Odds ratio; CI: Confidence interval.

**Table 5:** Incremental prognostic value of biomarker ratios beyond baseline severity scores for 28-day mortality prediction.

Metric	Model-1: Age + Sex + APACHE II + SOFA	Model-2: Model-1 + BAR + CAR + LAR + A/C	Δ (Model-2–Model-1)	*p*-Value
C-statistic (Harrell’s C)	0.823 (0.794–0.852)	0.872 (0.847–0.897)	+0.049	<0.001
NRI (continuous)	—	0.162 (0.094–0.230)	-	<0.001
NRI (categorical) *	—	0.141 (0.075–0.207)	-	<0.001
IDI	—	0.052 (0.031–0.073)	-	<0.001
Brier score	0.158	0.142	−0.016	-

* Risk categories for categorical NRI were defined as: low (<20%), intermediate (20–50%), and high (>50%) predicted risk.

## Data Availability

The data supporting the findings of this study are available from the corresponding author upon reasonable request. The data are not publicly available due to their use in ongoing analyses and model development.
